# Assessing cost and technical efficiency of HIV prevention interventions in sub-Saharan Africa: the ORPHEA study design and methods

**DOI:** 10.1186/s12913-014-0599-9

**Published:** 2014-11-29

**Authors:** Sergio Bautista-Arredondo, Sandra G Sosa-Rubí, Marjorie Opuni, Ada Kwan, Claire Chaumont, Jenny Coetzee, Jeanine Condo, Kumbutso Dzekedzeke, Omar Galárraga, Neil Martinson, Felix Masiye, Sabin Nsanzimana, Richard Wamai, Joseph Wang’ombe, on behalf of the ORPHEA study team

**Affiliations:** National Institute of Public Health (INSP), Division of Health Economics, Cuernavaca, Mexico; UNAIDS, Geneva, Switzerland; Perinatal HIV Research Unit, University of the Witwatersrand, Johannesburg, South Africa; National University of Rwanda, School of Public Health, Kigali, Rwanda; Dzekdzeke Research & Consultancy, Lusaka, Zambia; Brown University, Providence, USA; University of Zambia, Division of Economics, Lusaka, Zambia; Rwanda Biomedical Center, Kigali, Rwanda; Northeastern University, Boston, USA; University of Nairobi, School of Public Health, Nairobi, Kenya

**Keywords:** Cost, Economic evaluation, Technical efficiency, HIV, AIDS, Prevention, Testing, Male circumcision, PMTCT, Sex workers

## Abstract

**Background:**

Scaling up services to achieve HIV targets will require that countries optimize the use of available funding. Robust unit cost estimates are essential for the better use of resources, and information on the heterogeneity in the unit cost of delivering HIV services across facilities – both within and across countries – is critical to identifying and addressing inefficiencies. There is limited information on the unit cost of HIV prevention services in sub-Saharan Africa and information on the heterogeneity within and across countries and determinants of this variation is even more scarce. The “Optimizing the Response in Prevention: HIV Efficiency in Africa” (ORPHEA) study aims to add to the empirical body of knowledge on the cost and technical efficiency of HIV prevention services that decision makers can use to inform policy and planning.

**Methods/Design:**

ORPHEA is a cross-sectional observational study conducted in 304 service delivery sites in Kenya, Rwanda, South Africa, and Zambia to assess the cost, cost structure, cost variability, and the determinants of efficiency for four HIV interventions: HIV testing and counselling (HTC), prevention of mother-to-child transmission (PMTCT), voluntary medical male circumcision (VMMC), and HIV prevention for sex workers. ORPHEA collected information at three levels (district, facility, and individual) on inputs to HIV prevention service production and their prices, outputs produced along the cascade of services, facility-level characteristics and contextual factors, district-level factors likely to influence the performance of facilities as well as the demand for HIV prevention services, and information on process quality for HTC, PMTCT, and VMMC services.

**Discussion:**

ORPHEA is one of the most comprehensive studies on the cost and technical efficiency of HIV prevention interventions to date. The study applied a robust methodological design to collect comparable information to estimate the cost of HTC, PMTCT, VMMC, and sex worker prevention services in Kenya, Rwanda, South Africa, and Zambia, the level of efficiency in the current delivery of these services, and the key determinants of efficiency. The results of the study will be important to decision makers in the study countries as well as those in countries facing similar circumstances and contexts.

## Background

Improving the efficiency of the health sector is a growing international concern. The World Health Organization (WHO) estimates that 20%-40% of resources spent on health are wasted through inefficiency [1]. While more resources are required to ensure that coverage of essential health services is scaled up globally, significant improvements in health could be made with existing resources if countries only improved the efficiency of their health systems [1].

Optimizing HIV resources is imperative [2-4]. Despite more than a decade of significant increases in financing for HIV services in low- and middle-income countries (LMICs), the burden of HIV continues to exceed the funding available to address it. In 2012, almost US$ 19 billion were available for HIV prevention and treatment in LMICs [5]. Yet in the same year, an estimated 2.3 million people were newly infected with HIV, 1.6 million people died of AIDS-related causes, and although 9.7 million people received HIV treatment, this represented only 34% of those eligible under the most recent WHO HIV treatment guidelines [5].

Though there is wide agreement that additional resources are necessary to adequately address HIV in LMICs, new resources raised from international and domestic sources are unlikely to be sufficient in the immediate future [4,6,7]. Scaling up HIV services to achieve national and global targets will therefore require that countries get the most out of available funding by providing the optimal mix of evidence-based interventions targeted at appropriate populations and geographic areas to minimize new HIV infections and AIDS-related deaths (allocative efficiency) and by delivering the highest possible quality HIV services at the lowest feasible cost (technical efficiency) [2,3,8].

Information on the heterogeneity in the unit cost of delivering HIV services across facilities – both within and across countries – is critical to identifying and addressing inefficiencies. While higher costs can stem from facility and environmental characteristics as well as higher quality of the services provided, higher costs may result from inefficiencies that can be reduced [9]. A better understanding of the factors associated with high and low cost can help decision makers drive efficiency gains by addressing those issues.

There is limited information on the unit cost of HIV prevention services in sub-Saharan Africa where over 70% of people living with HIV reside [5]. Information on the heterogeneity of these costs within and across countries and determinants of this variation is even more scarce. Most empirical evidence on the efficiency of HIV prevention interventions comes from the “Prevent AIDS Network for Cost-Effectiveness Analysis” (PANCEA) project, which collected cost and output data between 2002 and 2004 for HIV testing and counselling (HTC), prevention of mother-to-child transmission (PMTCT), risk reduction for people who inject drugs, sex worker programs, treatment of sexually transmitted infections (STIs), and behavior change and communication [10,11]. The study which collected data from 206 programs in India, Mexico, Russia, South Africa, and Uganda revealed high levels of heterogeneity in average unit costs – even at the same number of units produced (scale) and within countries [11]. It found that on average, unit costs decreased with scale, but also that high levels of inefficiency were similar at any scale [11]. Since PANCEA, a number of empirical studies have found important differences in the unit cost of HTC across facilities and service delivery models in several countries including Kenya [12], Swaziland [12], Nigeria [13], Zambia [14], and Indonesia [15]. Studies comparing the unit cost of PMTCT across facilities in India [16] and Zambia [14] also found a great deal of variation. A study assessing the cost and efficiency of alternative male circumcision service delivery models in Kenya found modest differences [17]. Also, recent reviews of HIV intervention unit costs highlight the differences in unit costs of HIV prevention services across countries [9,18,19].

PANCEA and more recent related research have important limitations. These studies did not evaluate the quality of the services provided and therefore did not assess the possible role of service quality in the relationship between unit cost and scale. Another shortcoming of previous work is the limited insight provided on the determinants of the unit cost heterogeneity across service providers – both within and across countries. In addition, and unlike some more recent studies [12,17], PANCEA did not include direct observation of staff allocation of time. Previous evidence on health costing has shown that a large proportion of production costs are related to personnel [20]. Furthermore, there is evidence that self-reported time allocation (which is the method used in most studies) is biased and produces inaccurate cost estimates [21]. In the case of HIV services, determining staff allocation of time across HIV interventions is critical to accurately account for staff costs per intervention.

Designed to address these gaps, the “Optimizing the Response in Prevention: HIV Efficiency in Africa” (ORPHEA) study built on PANCEA and other past work on the cost and efficiency of HIV prevention services. Here we outline the ORPHEA study design and methods.

### Study aims and objectives

The ORPHEA study aims to add to the empirical body of knowledge on the cost and technical efficiency of HIV prevention services that decision makers can use to inform policy and planning. The study focused on four proven HIV prevention interventions that are central to national HIV responses of the most heavily affected countries in sub-Saharan Africa [4,22]. Included are general population interventions typically implemented in health facilities – HIV testing and counselling (HTC), prevention of mother-to-child transmission (PMTCT), voluntary medical male circumcision (VMMC), and services targeting specific groups usually implemented in non-clinical settings – HIV prevention services for female sex workers (FSWs) and male sex workers (MSWs).

The specific objectives of the ORPHEA study were:

1. To estimate the average cost per unit of output for each HIV prevention intervention, at the facility level.
2. To assess the level of efficiency for each service provider and for each HIV prevention intervention using the heterogeneity in average costs across facilities.
3. To explore the major determinants of efficiency for each HIV prevention intervention.

### Study setting

The ORPHEA study was implemented in Kenya, Rwanda, South Africa, and Zambia. These countries were purposively selected to have sufficient variability and representativeness across collected data (Table 1). The four countries represent a mix in terms of factors likely to impact HIV prevention including national population HIV prevalence, gross domestic product (GDP) per capita, per capita health expenditure, HIV funding sources, health system infrastructure, health information systems, and levels of HIV stigma and discrimination.

**Table 1:** **General information for countries included in the study (Kenya, Rwanda, South Africa, Zambia)**

	**Kenya**	**Rwanda**	**South Africa**	**Zambia**
Estimated HIV prevalence, adults (ages 15-49), 2012^1^	6.1	2.9	17.9	12.7
GDP per capita, 2012^2^	US$ 933	US$ 623	US$ 7314	US$ 1463
Health expenditure per capita, 2012^2^	US$ 45	US$ 66	US$ 645	US$ 96
Total population, 2012^2^	43,178,141	11,457,801	52,274,945	14,075,099
Number of people living with HIV (all ages), 2012^1^	1,600,000	210,000	6,100,000	1,100,000
% of total HIV expenditure that is international^3^	70	92	16	93
% of pregnant women living with HIV who received antiretrovirals for preventing mother-to-child transmission, 2013^4^	63	87*	90	76
% of overall coverage of circumcision among men aged 15-49, 2013^5^	91	13	46	13**

^1^Joint United Nations Programme on HIV/AIDS: *Global report: UNAIDS report on the global AIDS epidemic*. Geneva: Joint United Nations Programme on HIV/AIDS; 2013.

^2^The World Bank, World Development Indicators Online. http://data.worldbank.org/indicator Retrieved September 5, 2014.

^3^National AIDS Control Council, Kenya AIDS Response Progress Report, 2014; Rwanda Biomedical Center. Rwanda Global AIDS Progress Reports 2014; Republic of South Africa, Global AIDS Response Progress Rerport, 2012; National AIDS Council, Zambia Country Report, 2014.

^4^Joint United Nations Programme on HIV/AIDS: *The gap report*. Geneva: UNAIDS; 2014 (Kenya, South Africa, Zambia) and Joint United Nations Programme on HIV/AIDS: *Global report*. Geneva: Joint United Nations Programme on HIV/AIDS; 2013 (Rwanda) *Rwanda information from 2012.

^5^UNAIDS, AIDSInfo Online. http://www.aidsinfoonline.org/devinfo/libraries/aspx/Home.aspx Retrieved September 5, 2014. **Zambia data from 2009.

Given the study aim of providing evidence for decision-making, government interest in the study and receptiveness to recommendations expected to emerge were key elements taken into account during country selection. In each country, an essential preliminary phase of the study consisted of consultation with the relevant national government representatives: the National AIDS and STI Control Programme (NASCOP) and the National AIDS Control Council (NACC) in Kenya; the Rwanda Biomedical Center Institute of HIV/AIDS Disease Prevention and Control in Rwanda; the National Department of Health’s HIV and AIDS and STIs cluster in South Africa; and the National AIDS Council (NAC) in Zambia. In each country, we also consulted international partners such as the Joint United Programme on HIV/AIDS (UNAIDS), WHO, the World Bank, and the US Government; non-governmental organizations and civil society organizations; as well as public and private institutions involved in HIV service delivery. In Zambia, a specific committee – the Programme Efficiency Working Group – was set up to review and oversee ORPHEA and the World Bank “Zambia National HIV Programme Efficiency Study”.

## Methods/Design

### Study design

ORPHEA is a multi-country, cross-sectional observational study with three components: (1) estimation of the cost of providing HTC, PMTCT, VMMC, and HIV prevention for sex workers; (2) assessment of facility and contextual characteristics including possible determinants of efficiency; and (3) appraisal of process quality for the facility-based interventions (HTC, PMTCT, and VMMC) [23].

ORPHEA collected information at three levels. Firstly, at the district level, information was collected on district-level inputs to the facility delivery of HIV prevention services and their prices, along with district-level characteristics and factors likely to influence demand for HIV prevention services within a district. Secondly, at the facility level, the study collected data on the inputs to HIV prevention service production and their prices, outputs produced along the cascade of services, and a variety of facility-level characteristics and contextual factors. Thirdly, data were collected from individuals including information on process quality for HTC, PMTCT, and VMMC gathered from providers and clients through provider vignettes and client exit interviews, respectively.

The ORPHEA study methods were consistent across the four countries in terms of design, methods, and data collection tools in order for the results to be comparable across countries as well as within them. The study team made only minor adaptations to study questionnaires and implementation to take into account differences in context, service delivery, and geographic distribution of services. Common survey details across the countries that required adaptation included the output indicators for each intervention (in all countries, these were extracted from monthly summary reports generated by facilities for district-level reporting for HTC, PMTCT, and in some instances for VMMC; in other cases, VMMC indicators were retrieved from non-governmental implementers), job titles and salary grades, and facility types and levels of the health system. The sex worker interventions covered varied slightly by country because of the availability of organizations delivering HIV prevention for this key population in the different countries. HIV prevention interventions for male sex workers were added in Kenya since per the national guidelines for sex workers, they have been identified as having an increased risk for HIV and STIs [24]. HIV prevention interventions for female sex workers were not included in Zambia because no programs could be identified. In South Africa, the only services targeted at sex workers that could be identified were operated by the trucking industry. In addition to survey content and intervention mix, adjustments to the data collection schedule were made to ensure that data collection occurred at the times when the interventions were offered (e.g., when the VMMC program was being implemented).

### Sample size and site selection

**Figure 1:**
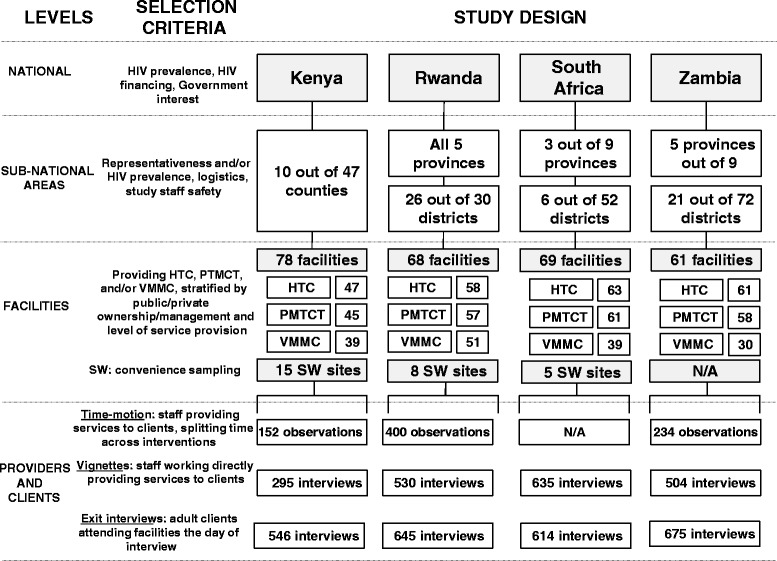
Sampling strategy and sample sizes by ORPHEA study component*. *This figure includes all sites visited by the study’s fieldwork teams but excludes facilities initially selected but replaced during fieldwork.

For each country sample, multistage sampling was used to select sub-national areas and health facilities providing HTC, PMTCT, and VMMC. The study team used a number of rigorous and replicable sampling techniques to identify the sub-national areas, the facilities, and the individuals included in the ORPHEA study. Sub-national areas were selected purposively based on local HIV prevalence and other logistical considerations including study staff security (Figure 1).

A health facility sampling frame was created for all selected sub-national areas with information on the provision of HTC, PMTCT, and VMMC stratified by ownership/management (e.g., for-profit and not-for-profit service providers, and faith-based facilities) and level of service provision (e.g., hospitals, maternity clinics, primary care clinics, and dispensaries). Facilities were randomly sampled within these strata using probability proportional to size with preference given to integrated sites providing more than one HIV prevention service of interest versus stand-alone sites providing only one of the interventions studied.

The sample size for the facility-based interventions (HTC, PMTCT, and VMMC) was calculated to identify the minimal number of facilities necessary to detect statistically significant associations between average unit cost per facility and the determinants of efficiency. Sample size calculations were informed by existing data – the PANCEA study results on the correlation between the scale of HTC programs and their unit cost. The PANCEA study found that the correlation coefficients between the annual number of HTC clients and the cost per HTC were as follows: -0.45 in Mexico, -0.54 in Uganda, -0.53 in Russia, -0.91 in India, and -0.57 in South Africa [10]. The ORPHEA study was therefore powered to detect correlations at least as important as the correlation between HTC scale and cost found in Mexico in the PANCEA study – i.e., the weakest correlation identified. This translated into a target sample size of 40 sites per intervention (HTC, PMTCT, and VMMC) per country.

For sex worker HIV prevention service providers, the sample size was derived differently. Given that the number of programs providing services for this group in each country was limited and that they were provided in very different formats to the other HIV prevention services, the maximum number of sites was set at 15. A review of all available sites was undertaken and those responding to the inclusion criteria were selected into the study. Provision of HIV testing and HIV-related behavior change interventions were the main inclusion criteria.

The numbers of facilities included for each country are summarized in Figure 1, as is the number of sites included for each of the four interventions studied. The relevant sample sizes and the algorithms used to select the staff for the time motion and health provider vignettes and the clients for the client exit interviews are also specified in Figure 1.

### Data collection instruments

**Figure 2:**
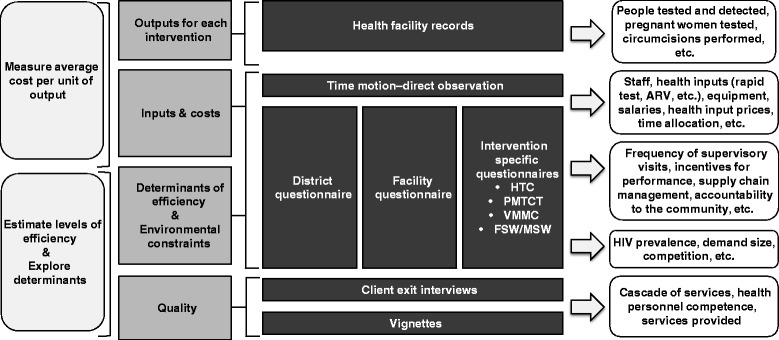
**ORPHEA study questionnaires.**

A standardized set of ORPHEA study survey instruments was developed, some of them based on the instruments used in the PANCEA project [10,11]. All instruments were piloted in each of the four countries and minor adaptions were made for each country setting, including additional options for question responses, categories of personnel, facility types, and levels of the health system. Figure 2 and Table 2 provide an overview of the data collection instruments used in ORPHEA along with the categories of information collected with each one.

**Table 2:** **Summary of ORPHEA study questionnaires**

**Name of questionnaire**	**Category of information**	**Mode of administration**	**Type of typical respondent**	**Data sources**	**Examples of questions obtained**
District	Determinants of efficiency and environmental constraints; inputs & costs	Face-to-face interview, data extraction from records when needed	District Officer, District HIV/AIDS Coordinator, Head accountant, Head administrator	National database or reports, staff records, district records	HIV prevalence in the catchment area, demand size, competition, etc.
Facility	Determinants of efficiency and environmental constraints; inputs & costs	Face-to-face interview, data extraction from records when needed	Facility in-charge, HIV/AIDS services in-charge	Accounting records, staff payroll, performance reports & records, HR records, inventories	Frequency of supervisory visits, incentives for performance, supply chain management, accountability to the community, equipment, salaries, health input prices
Intervention specific (HTC, VMMC, PMTCT, F/MSW)	Determinants of efficiency and environmental constraints; inputs & costs	Face-to-face interview	Service coordinator, HIV/AIDS coordinator	Medical and pharmacy records, logbooks or registers	Characteristics of service provision
Time motion - Direct observation	Inputs (staff time allocation)	Direct observation of providers	Providers directly providing services to clients	N/A	Staff time allocation
Provider vignettes	Quality	Face-to-face interview	Providers directly providing services to clients	N/A	Cascade of services, health personnel competence, services provided
Exit interviews	Quality	Face-to-face interview	Adult clients attending the facility	N/A	Cascade of services, health personnel competence, services provided
Health facility records	Outputs for each intervention	Data extraction from records	M&E Officer, Facility in-charge	Health facility records	People tested and detected, pregnant women tested, circumcisions

Data collection tools included data abstraction forms for capturing relevant quantitative information from program records and reports, particularly for HIV service outputs (types and number of services provided) and for all relevant inputs used to produce them. Other data sources included: reporting forms, electronic databases, electronic or written medical records, logbooks, registers, receipts, stock cards, facility accounting records, payroll records, and performance reports. For other data items, namely service quality and staff time allocation, structured interview and observational forms were used.

### Data collection

The research team at the National Institute of Public Health (INSP), Mexico oversaw the overall ORPHEA study. Implementation of the study in Kenya, Rwanda, South Africa, and Zambia was coordinated by researchers from the following collaborating research institutions: University of Nairobi, Brown University, and Northeastern University (Kenya); University of Rwanda School of Public Health and Rwanda Biomedical Center Institute of HIV/AIDS Disease Prevention and Control (Rwanda); Perinatal HIV Research Unit, University of the Witwatersrand, (South Africa); and the University of Zambia Division of Economics (Zambia).

In each country, data collectors were selected based on previous experience and credentials and underwent a 2-3 week training on the study instruments and the methods required to properly administer the data collection tools. Training included role playing in which fieldwork teams administered the interview instruments to each other along with applying the data abstraction instruments on actual data obtained from facilities delivering HIV prevention interventions. A pilot was carried out after the training and was followed by a debriefing session during which final decisions were made and instructions were given to the fieldwork teams.

The instruction of data collectors in all countries also included training sessions on human subjects research. These sessions stressed the importance of obtaining informed consent, the voluntary nature of participation in the study, and the importance of transparency and honesty in all interactions with study subjects.

All study tools were administered in English, with the exception of the exit interview questionnaire, which was translated and administered in English as well as 12 other languages: Kiswahili in Kenya; French and Kinyarwanda in Rwanda; Sesotho, Siswati, and Zulu in South Africa; and Bemba, Kaonde, Lozi, Lunda, Nyanja, and Tonga in Zambia.

Data collection instruments were administered to district officers, facility officers-in-charge, or other management staff, service providers, and clients. Leading staff at all locations were asked to consent to data collection and direct observation. Other staff interviewed or observed were also asked for written consent. For the time-motion component and the exit interviews, the researchers asked for informed consent from providers and patients, respectively. All instruments were administered in a quiet private place after obtaining informed consent from study participants.

Data collection was staggered by country between October 2012 and December 2013. Data were collected in Kenya from October 2012 – June 2013; in Zambia from December 2012 – July 2013; in South Africa from March 2013 – August 2013; and in Rwanda from July 2013 – December 2013.

### Measuring inputs and costs

In order to compute total cost of production, a micro-costing approach was used in which both the quantity and the unit price of the essential inputs were collected. Input and cost data were collected retrospectively by month for the entire previous calendar year, and for the month prior to data collection. These data were collected at the facility-level as well as at the district-level through the district questionnaire, the facility questionnaire and the intervention-specific questionnaires. The ORPHEA instruments were designed to assemble five categories of costs: personnel, recurrent inputs and services, capital, and training and supervision. We collected cost data irrespective of which funder financed the HIV prevention services under consideration. Many clinics received inputs from a variety of sources. All donated inputs were valued at their opportunity costs determined by local market prices, adopting economic rather than financial costing [25].

### Measuring time motion

Time motion, the gold standard tool in measuring staff allocation of time through direct observation [21,26], was implemented at facilities where HIV prevention interventions were integrated (i.e., delivered in facilities providing more than one HIV prevention service). Data collectors selected and observed up to six providers per integrated facility (see Figure 1). For each of the selected providers, data were collected on activities performed including time spent on administrative work, meetings, and breaks and the duration of time spent on each activity for three to four continuous hours. The Hawthorne effect was minimized by coding activities at the level of the intervention and not coding more specific activities, informing providers that observers would not evaluate their work, and making all observations outside of facility observation rooms [27]. Observing providers outside their work space respected ethical guidelines, as we maintained patient confidentiality and did not interfere with the providers’ work or reduce the time providers spent with patients.

### Measuring outputs

Output data reflecting health service utilization (production) were also collected monthly and retrospectively for the entire calendar year, and for the month prior to data collection. These data were collected at the facility level through the health facility records questionnaire, and if not available, from the district health information system. Information was collected on multiple outputs corresponding to steps along the cascade of services for HTC and PMTCT (see Table 3).

**Table 3:** **Simplified service cascade indicators used to assess outputs for HTC, PMTCT, and VMMC services at each facility**

**Intervention**	**Indicator along simplified service cascade (numbers per month)**
HTC	Clients tested
	Clients tested and positive
PMTCT (women and babies)	Clients tested
	Clients tested and positive
	Clients on HAART
	Exposed infant
	Exposed infant tested
	HIV negative exposed infant
VMMC	VMMC performed

### Measuring process quality

Provider vignettes and client exit interviews were the primary process quality measures of the three facility-based HIV prevention services – HTC, PMTCT, and VMMC. In Rwanda, exit interviews and vignettes were also implemented for the facility-based component of the sex worker intervention. The structures of both of these instrument types were designed based on both the national guidelines in each country, if available, and WHO guidelines and guidance documents. Provider vignettes and client exit interviews were conducted during the time period when the field teams were present at facilities to assess service quality measures for estimating provider competence and performance [23]. Logistically, exit interviews were planned for the days in which each specific intervention was offered in order to fulfill the interview quota set for each intervention (described below), and vignettes were planned for the time periods or days in which providers offering the service were less busy in order to not disrupt their work.

As a measure of provider competence, the vignette questionnaire assessed the extent to which existing guidelines about HIV prevention services were adhered to by health providers who offered HTC, PMTCT, or VMMC services. Health workers who had contact with HTC, PMTCT, and VMMC clients at the facility on the day of data collection were considered for vignette interviews with up to five providers interviewed for each intervention in a given facility (see Figure 1). The vignette presented the respondent with a scenario capturing a hypothetical HTC, PMTCT, or VMMC client. Each respondent was asked about how they would take a medical history, perform a physical examination (if applicable), order appropriate laboratory or imaging tests, assess disease severity and functionality, and prescribe treatment (if applicable).

As a measure of provider performance, exit interviews assessed the process quality of HTC, PMTCT, and VMMC services provided at the facility from the client’s perspective. The exit interview module was directed at patients attending the facility on the day of data collection for HTC, PMTCT, and VMMC services with up to five client interviews to be completed for HTC and VMMC services each and up to eight interviews to be completed for PMTCT. More interviews were assigned to PMTCT to capture the larger service window beginning with antenatal to postnatal care. Patients were identified in the waiting area, near the exit of the facility or of the service site of interest. The exit interviews were designed to collect information on the process quality of the visit from the perspective of the patient following the same structure and including the same components as the vignettes, the reasons for visiting the facility, access to health services, referrals from other facilities, costs incurred and what the HIV prevention services received by the patient consisted of [23].

### Measuring facility-level and contextual factors related to efficiency

The district, facility and intervention-specific questionnaires collected information on a variety of facility and contextual factors. Constraints were the first category of factors of interest. Constraints are factors that can modify the cost of delivering HIV prevention services but do not depend on a facility’s performance. They are exogenous to facility-level decisions. We included both institutional and contextual constraints. Institutional constraints included those policies and regulations instituted above the facility-level. Contextual constraints included such characteristics as the size of the target population and the size of the population using the services.

The second category of facility and contextual factors of interest were the determinants of efficiency. Determinants are characteristics that also influence the cost of the delivery of the service and they depend on a facility’s performance. Included as determinants were facility-level characteristics such as organizational structure, management, governance, decision-making processes, funding sources, training, supervision, incentives, and accountability.

An important mechanism to incentivize improvements in the production of health services is the accountability structure in place. Three main accountability channels through which information can affect learning outcomes have been suggested in the field of education: increasing choice, participation, and voice [28]. These three mechanisms can be applied to accountability in health service provision. Hence, inputs to identify the degree of choice and participation and voice of service users regarding HIV health services were included in the questionnaires to capture the main accountability elements in facilities.

### Data management

Except the exit interview and the time motion instruments, all the other questionnaires were administered on pre-programmed CSPro databases based on the Computer-Assisted Paperless-Interview (CAPI) technology (US Bureau of the Census). Data from each data collection team were uploaded daily to a secure internet cloud service folder. This allowed for regular back up of data to facilitate almost real-time quality checks. A data manager reviewed the data uploaded daily by each team and assessed its completeness and consistency using a standardized checklist. The data manager also merged and exported the databases into formats compatible with STATA data analysis software (StataCorp LP, College Station, Texas, USA) to allow a second data manager based in Mexico to further examine the data every few days. Together, these procedures allowed for the assessment of the completeness and quality of the data on an on-going basis, for providing feedback to each team on the quality of their work, and going back to data sources and informants as needed.

In the case of the exit interviews and the time motion instruments, the study teams followed strict data confidentiality procedures. Hard copies of data collection materials did not identify patients; they had identifiers for health facilities and districts. After the research teams visited health facilities and conducted patient interviews, the completed survey forms were kept in sealed envelopes until they were collected by a supervisor. Supervisors transported the forms to the country coordinating research institution where they were stored in a locked, secure cabinet, with limited access by specified individuals only.

### Data analysis

At the time of writing, data analysis was in process; study findings will be published in subsequent papers. Total production costs will be calculated for each intervention across the sampled facilities in each country. Costs for personnel, recurrent supplies, operating costs, and capital will be summed for the year of observation for each intervention. Average costs per intervention will be calculated as will the average costs at each step of the service cascade for each intervention.

For each country, the relationship between average unit cost and scale of production (i.e., the number of clients) as well as process quality indicators will be assessed for each facility type using correlations. And to further examine this relationship, for each service, intervention costs will be regressed against scale of production adjusted for service quality, HIV prevalence at local level, facility type and management variables including governance, accountability, supervision, monitoring, and incentives [29].

For the comparative costing analyses, efficiency will be defined as the minimum average cost per unit of output at each stage of the cascade for each HIV prevention intervention at given levels of scale, scope, and other exogenous characteristics. The comparison of average unit cost per intervention will provide a picture of the levels of efficiency in the production of HIV prevention services across facilities and across countries.

In addition, inefficiency in the sample sites will be estimated using rigorous methodological alternatives. First, we will estimate the production technology using cost functions [30-32]. Second, we will also use stochastic frontier analysis (SFA) [33], data envelopment analysis (DEA) [34], and a recent hybrid approach of these two techniques proposed by Wagstaff and Wang [35]. While SFA is a parametric technique which uses a regression model to estimate the production or cost frontier, DEA is a non-parametric method that uses linear programming techniques to construct a piecewise frontier over the observed data. The focus of the analyses will be on producing evidence that can be translated into practical recommendations to increase the efficiency of HIV preventions services for policy makers.

### Ethical clearance

Ethical clearance for this study was granted by the National Institute of Public Health (INSP), Mexico Ethics Committee (approval number 1150); Kenyatta National Hospital and University of Nairobi Ethics and Research Review Committee (approval number KNH-ERC/MOD/161); Northeastern University Human Subject Research Protection (reference number 12-07-20); Rwanda Biomedical Center National Health Research Committee (approval number NHRC/2012/PROT/0036); University of the Witwatersrand, Johannesburg Human Research Ethics Committee (approval number MI20832); and the University of Zambia Biomedical Research Ethics Committee (reference number 025-06-12).

## Discussion

Scaling up HIV services to achieve national and global targets will require that countries make concerted efforts to optimize the use of available funding. Information on the unit cost of services is critical to identifying and addressing the inefficiencies impeding the delivery of the highest possible quality HIV services at the lowest feasible cost. Empirical data on the cost and technical efficiency of HIV prevention services in the countries most impacted by HIV are scarce and can provide guidance to achieve this goal. The ORPHEA study built on past studies while addressing some of the key limitations of previous work and is one of the most comprehensive studies on the cost and technical efficiency of HIV prevention interventions to date. The results of the study will be important to decision makers in the study countries as well as those in countries facing similar circumstances and contexts.

The ORPHEA study has important strengths. It is a multi-country study including four countries that differ in terms of factors likely to impact HIV prevention (e.g., national population HIV prevalence and GDP per capita): Kenya, Rwanda, South Africa, and Zambia. With 304 service delivery sites included across the countries ranging from 61 in Zambia to 93 in Kenya, the sample size of the study is large. Moreover, ORPHEA applied a robust methodological design and data collection tools to collect comparable information to estimate the cost of HTC, PMTCT, VMMC, and sex worker prevention services, the level of efficiency in the current delivery of these services, and the key determinants of efficiency – both within and across countries. In contrast to earlier work, information was collected on multiple outputs for each intervention corresponding to steps along the cascade of services for HTC and PMTCT. In addition to providing information of the cost per service along the cascade, we hypothesize that levels of attrition along the cascade may capture a dimension of service quality with higher levels of attrition along the cascade suggesting lower levels of quality. Also unlike earlier studies, through provider vignettes and client exit interviews, ORPHEA captured an assessment of process quality for the three facility-based HIV prevention services – HTC, PMTCT, and VMMC. Finally, building on previous work, direct observation of staff allocation of time was a central component of the study addressing another common limitation of many previous studies.

ORPHEA also has some limitations. Though systematic sampling was used, the selected samples are unlikely to be nationally or provincially representative mainly because a first stage in the sampling strategy included a purposeful selection of states or provinces. In addition, we had particular difficulty identifying sex worker HIV prevention service providers. Although all identified sites responding to the selection criteria were included in the study, some of the sex worker HIV prevention country samples did not reach the goal of 15 sites due to the low number of qualifying sex worker programs. Some additional limitations in measurement are as follows. Every effort was made to capture staff costs as accurately as possible through time-motion methods. Yet there are limitations to these methods as well including the potential bias introduced by the Hawthorne effect and the possibility that the days and hours of staff moments monitored are not representative of the entirety of staff moments in a given year. Assessment of service quality is critical to studies like this one and the inclusion of process quality measurement is an important strength of ORPHEA compared to previous work but process quality is only one aspect of quality. Lastly, there is limited research on the essential management characteristics associated with well-functioning facilities in LMICs. We made every attempt to be comprehensive given the information available but it is possible that some key aspects of the management of HIV prevention services that are related to efficiency were not captured in our study. Future studies can build on our efforts, as we did on previous ones, to expand our knowledge on this topic.

## Abbreviations

DEA: Data envelopment analysis
HTC: HIV testing and counselling
INSP: National institute of public health, Mexico
PANCEA: Prevent AIDS network for cost-effectiveness analysis
PMTCT: Prevention of mother-to-child transmission
ORPHEA: Optimizing the response in prevention: HIV efficiency in Africa
SFA: Stochastic frontier analysis
STI: Sexually transmitted infection
SW: Sex worker
VMMC: Voluntary medical male circumcision
